# Optimization of Swertiamarin and Isogentisin Extraction from *Gentiana lutea* L. Leaves by Response Surface Methodology

**DOI:** 10.3390/plants14162538

**Published:** 2025-08-15

**Authors:** Katarina Šavikin, Miloš S. Jovanović, Gordana Zdunić, Jelena Živković, Dušanka Kitić, Dubravka Bigović, Teodora Janković

**Affiliations:** 1 Institute for Medicinal Plants Research “Dr. Josif Pančić”, Tadeuša Košćuška 1, 11000 Belgrade, Serbia; ksavikin@mocbilja.rs (K.Š.); gzdunic@mocbilja.rs (G.Z.); jzivkovic@mocbilja.rs (J.Ž.); dbigovic@mocbilja.rs (D.B.); tjankovic@mocbilja.rs (T.J.); 2 Department of Pharmacy, Faculty of Medicine, University of Niš, Boulevard Dr. Zorana Đinđića 81, 18000 Niš, Serbia

**Keywords:** yellow gentian, secoiridoids, xanthones, ultrasound-assisted extraction, extraction parameters, storage stability

## Abstract

Leaves of *Gentiana lutea* L., traditionally used for treating heart disorders, represent a sustainable and underutilized source of bitter secoiridoids and xanthones, also found in *Gentianae radix*—an official herbal drug derived from the same, protected species. As root harvesting leads to the destruction of the plant, using the more readily available leaves could help reduce the pressure on this endangered natural resource. This study aimed to optimize the ultrasound-assisted extraction of the secoiridoid swertiamarin and the xanthone isogentisin from *G. lutea* leaves using response surface methodology (RSM). Subsequently, the stability of the bioactive compounds (swertiamarin, gentiopicrin, mangiferin, isoorientin, isovitexin, and isogentisin) in the optimized extract was monitored over a 30-day period under different storage conditions. The influence of extraction time (5–65 min), ethanol concentration (10–90% *v*/*v*), liquid-to-solid ratio (10–50 mL/g), and temperature (20–80 °C) was analyzed at five levels according to a central composite design. The calculated optimal extraction conditions for the simultaneous maximization of swertiamarin and isogentisin yields were 50 min extraction time, 30% *v/v* ethanol concentration, 30 mL/g liquid-to-solid ratio, and 62.7 °C extraction temperature. Under these conditions, the experimentally obtained yields were 3.75 mg/g dry weight for swertiamarin and 1.57 mg/g dry weight for isogentisin, closely matching the RSM model predictions. The stability study revealed that low-temperature storage preserved major bioactive compounds, whereas mangiferin stability was compromised by elevated temperature and light exposure. The established models support the production of standardized *G. lutea* leaf extracts and may facilitate the efficient separation and purification of their bioactive compounds, thereby contributing to the further valorization of this valuable plant material.

## 1. Introduction

The genus *Gentiana* L., the largest within the Gentianaceae family with approximately 400 species, is primarily distributed in alpine regions of temperate zones worldwide, particularly in Europe, Southeast Asia, and North America. It comprises many medicinal plants well known in traditional medicine with a long history of use. Due to their therapeutic potential, *Gentiana* species have attracted considerable scientific interest in phytochemical and pharmacological research over recent decades. As a result, nearly 600 metabolites exhibiting around 20 different biological activities have been identified from members of this genus. Among them, xanthones, flavonoids, iridoids, and triterpenoids are the major classes of secondary metabolites [1,2,3]. A notable challenge in the development of pharmaceutical preparations from *Gentiana* species is the instability of bitter iridoid compounds (including secoiridoids) and xanthones, observed both during the processing of plant material [4] and storage [5,6].

Numerous in vitro, in vivo, and clinical studies have demonstrated the therapeutic potential of *G. lutea* extracts and their bioactive constituents as anti-inflammatory, antioxidant, antimicrobial, anti-atherosclerotic, anti-obesogenic, neurotrophic, gastroprotective, and anti-genotoxic agents, thereby highlighting their pleiotropic properties [7]. According to the EMA/HMPC monograph, preparations of *Gentianae luteae radix* are traditionally used for the symptomatic treatment of temporary loss of appetite and mild gastrointestinal disorders [8]. Besides its medicinal use, gentian root is traditionally used in many European countries to prepare bitter alcoholic beverages, with secoiridoids as the main bitter principles. However, the preparation of such products requires harvesting large amounts of roots from plants that are at least five years old. Due to the slow natural regeneration of *G. lutea* populations, harvesting represents a significant threat to its sustainability. As a result, the collection of gentian root is subject to strict national and regional regulations, and the species is legally protected in several European countries [9]. Previous phytochemical studies have justified the use of not only the root but also the aerial parts of *G. lutea* as medicinal material [9,10,11,12]. The use of leaves as an alternative source offers possibilities for the sustainable use of *G. lutea*. In addition to their potential as a substitute for the root, primarily in the treatment of gastrointestinal disorders, *G. lutea* leaves have also been traditionally used for certain cardiovascular conditions, such as angina pectoris [10].

In recent decades, ultrasound-assisted extraction (UAE) has found broad application in the pharmaceutical and food industries due to its efficiency and lower processing costs [13]. This method enables the extraction of bioactive compounds in a short time, under mild temperatures, and with reduced energy and solvent consumption [14]. Consequently, UAE is recognized as a green extraction method appropriate for thermo-sensitive compounds [15]. UAE relies on the phenomenon of ultrasonic cavitation, where ultrasound waves generate alternating high- and low-pressure cycles within the liquid medium, leading to the formation of cavitation bubbles [13,14]. The collapse of cavitation bubbles (implosion) generates localized mechanical and thermal stresses that act on plant tissue, leading to cell wall disruption. This facilitates the release of intracellular bioactive compounds into the surrounding solvent, driven by a mass transfer gradient. The extraction process may involve one or more concurrent mechanisms, including fragmentation, erosion, structural disintegration, capillary action, and sonoporation. Together, these mechanisms enhance extraction efficiency by promoting cellular breakdown and accelerating mass transfer [15]. The efficiency of the UAE process depends on numerous operational parameters, including ultrasonic power, frequency, duty cycle, type of solvent, liquid-to-solid ratio, temperature, solvent pH, and extraction time [14]. Therefore, the optimization of UAE conditions is required, as variations in plant matrices and the physicochemical properties of target compounds may necessitate an individualized approach for each plant material. Response surface methodology (RSM) is one of the most commonly used optimization techniques for the extraction of bioactive compounds from plant material. Unlike conventional optimization methods, it allows for a structured experimental approach to evaluate the effects of individual and interacting factors. This enables rapid optimization with minimal resource consumption [14,15].

Previous studies have applied RSM to optimize the extraction of bioactive compounds from *G. lutea* root using UAE and heat-assisted extraction [16,17]. However, to the best of our knowledge, no such optimization has been conducted for the extraction of bioactive compounds from *G. lutea* leaves. To address this gap, the present study aimed to optimize the UAE of two marker compounds from *G. lutea* leaves: the secoiridoid swertiamarin and the xanthone isogentisin, representing secoiridoids and polyphenols, respectively. The optimization was carried out using RSM based on a central composite design (CCD), with extraction time, ethanol concentration, liquid-to-solid ratio, and extraction temperature as the process variables. Furthermore, the storage stability of bioactive compounds in *G. lutea* leaves was assessed under various conditions. The conclusions drawn from this study offer optimized processing parameters for the efficient recovery of bioactive compounds from *G. lutea* leaves by UAE, potentially serving practical interests in the development of pharmaceutical and nutritional products. To the best of our knowledge, this is the first study to apply RSM–CCD for optimizing the UAE of bioactive compounds from *G. lutea* leaves, followed by an assessment of their storage stability, representing an initial step toward the valorization of this plant material for pharmaceutical and nutritional applications.

## 2. Results

### 2.1. Model Fitting

The yields of swertiamarin and isogentisin extracted under the conditions set by the RSM-CCD design are presented in Table 1.

Analysis of variance (ANOVA) was used to evaluate the adequacy of the fitted models and to identify the factors and their interactions that significantly affected the extraction efficiency of swertiamarin and isogentisin. The ANOVA results are presented in Table 2. Based on the *p*-values, the significance of model terms was classified as follows: non-significant (*p* > 0.05), significant (0.05 > *p* > 0.01), highly significant (0.01 > *p* > 0.001), and remarkably significant (0.001 > *p*). The predicted quadratic polynomial models for both evaluated responses demonstrated high statistical significance, with *p*-values lower than 0.0001. The coefficient of determination (R^2^) was 0.71 for swertiamarin and 0.84 for isogentisin, indicating a satisfactory agreement between the observed and predicted values. A non-significant lack-of-fit (*p* > 0.05) indicates that the models provide an adequate representation of the relationship between the investigated variables and the observed responses.

### 2.2. Influence Analysis

#### 2.2.1. Influence of Independent Variables on the Extraction Efficiency of Swertiamarin

**Figure 1:**
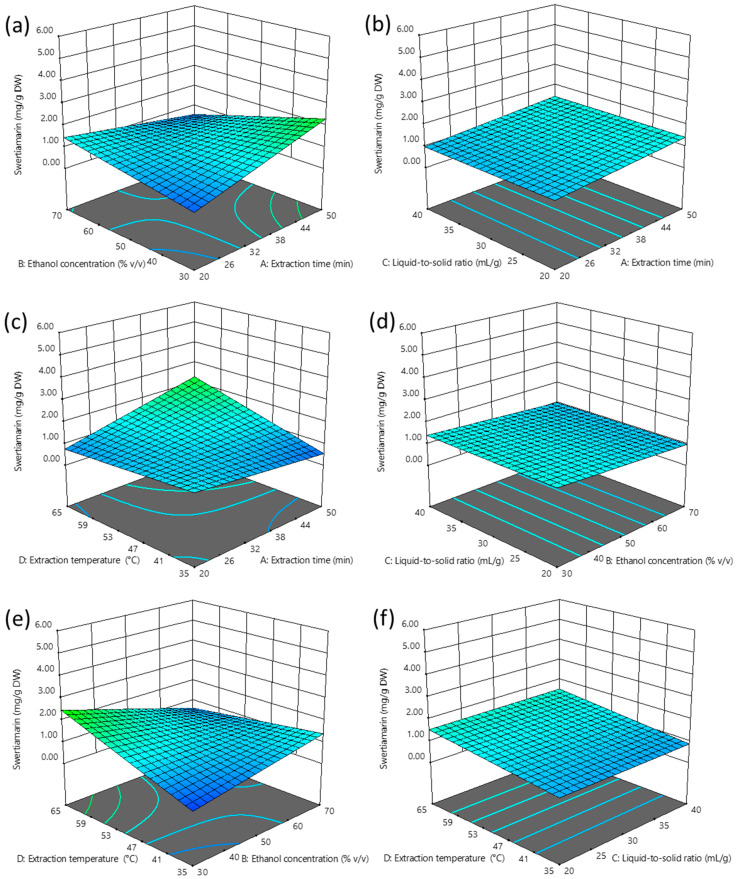
Three-dimensional RSM plots illustrating extraction yield of swertiamarin as the dependent variable and UAE process parameters as independent variables (**a**–**f**). The levels of variables not displayed in the plots were fixed at their central values.

**Figure 2:**
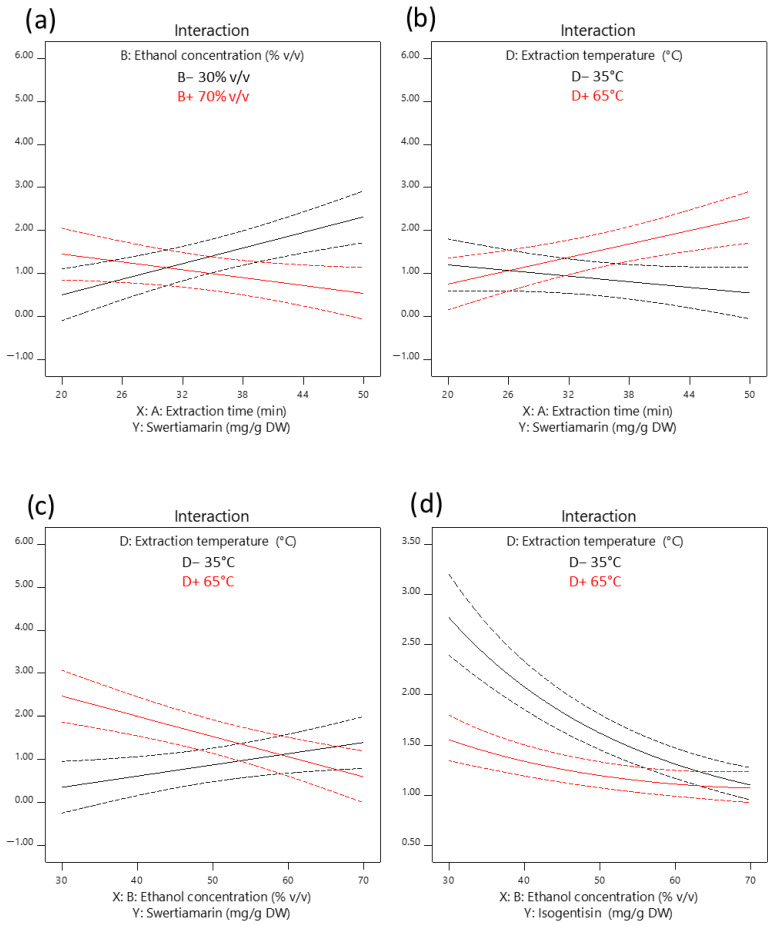
Interaction RSM plots of the terms included in the UAE models, illustrating the influence of extraction conditions on the extraction yield of swertiamarin (**a**–**c**) and isogentisin (**d**). The levels of variables not displayed in the plots were fixed at their central values. Dashed lines indicate the 95% confidence intervals.

Figure 1a–f and Figure 2a–c present three-dimensional and interaction RSM plots, respectively, of swertiamarin extraction yield as a function of UAE conditions.

The extraction yield of swertiamarin, the dominant secoiridoid in gentian leaves, ranged from trace amounts (close to 0) up to 5.17 mg/g DW. Such a pronounced difference in swertiamarin extraction efficiency highlights the importance of selecting optimal UAE process parameters to ensure maximal recovery of target compounds from the plant material. The highest yield of swertiamarin was achieved at an extraction time of 50 min, ethanol concentration of 30% *v*/*v*, liquid-to-solid ratio of 40 mL/g, and extraction temperature of 65 °C. Conversely, the lowest yield occurred at 20 min extraction time, 30% *v/v* ethanol concentration, 40 mL/g liquid-to-solid ratio, and 35 °C extraction temperature.

According to the results of the ANOVA (Table 2), the extraction yield of swertiamarin was significantly affected by the extraction temperature as a positive linear term (+D), antagonistic interactions between extraction time and ethanol concentration (−AB), and between ethanol concentration and extraction temperature (−BD), as well as a synergistic interaction between extraction time and temperature (+AD). A predictive model equation for swertiamarin extraction yield, expressed in terms of coded variables and obtained by excluding non-significant terms while retaining those necessary to maintain model hierarchy, was as follows:

Swertiamarin (mg/g DW) = 1.20 + 0.22 A − 0.21 B + 0.33 D − 0.68 AB + 0.55 AD − 0.73 BD

The calculated optimal extraction conditions for obtaining the highest swertiamarin yield (3.93 mg/g DW) include 50 min of extraction time, 30% *v/v* ethanol, a liquid-to-solid ratio of 40 mL/g, and an extraction temperature of 65 °C.

#### 2.2.2. Influence of Independent Variables on the Extraction Efficiency of Isogentisin

**Figure 3:**
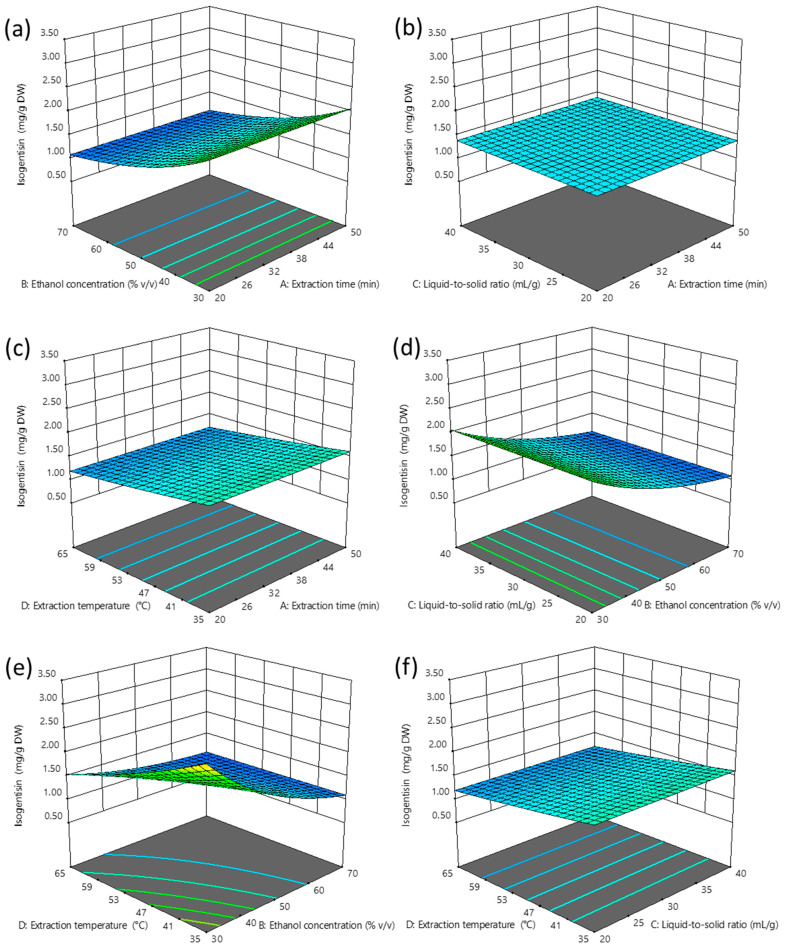
Three-dimensional RSM plots illustrating extraction yield of isogentisin as the dependent variable and UAE process parameters as independent variables (**a**–**f**). The levels of variables not displayed in the plots were fixed at their central values.

Figure 3a–f and Figure 2d present three-dimensional and interaction RSM plots, respectively, of isogentisin extraction yield as a function of UAE conditions.

The extraction yield of isogentisin obtained from gentian leaves using UAE ranged from 0.83 to 3.32 mg/g DW. The highest yield was observed under the same extraction conditions at which swertiamarin yield was the lowest: 20 min of extraction, 30% *v/v* ethanol, a liquid-to-solid ratio of 40 mL/g, and a temperature of 35 °C. In contrast, the lowest isogentisin yield was recorded at 20 min, 70% *v/v* ethanol, a liquid-to-solid ratio of 20 mL/g, and an extraction temperature of 35 °C.

Based on the ANOVA results (Table 2), the extraction yield of isogentisin was primarily influenced by ethanol concentration, which exhibited a significant negative linear effect (−B) and a positive quadratic effect (+B^2^). In contrast to the extraction efficiency of swertiamarin, extraction temperature had a significant negative linear effect (−D) on the extraction yield of isogentisin. Among the interaction effects, only the interaction between ethanol concentration and extraction temperature was statistically significant, showing a synergistic effect (+BD), which also contrasts with the previously observed antagonistic effect of the same interaction on the extraction yield of swertiamarin. A predictive equation for isogentisin extraction yield, expressed in terms of coded variables and obtained by excluding non-significant terms, was as follows:

Log_10_ (Isogentisin) (mg/g DW) = 0.14 − 0.14 B − 0.07 D + 0.06 BD + 0.03 B^2^

The optimal extraction conditions predicted for achieving the highest isogentisin yield (2.73 mg/g DW) are 50 min of extraction time, 30% *v/v* ethanol concentration, a liquid-to-solid ratio of 20 mL/g, and an extraction temperature of 35 °C.

### 2.3. Optimization of Process Parameters

By applying the desirability function to both target compounds (swertiamarin and isogentisin), the optimal UAE conditions from gentian leaves were identified as follows: extraction time of 50 min, ethanol concentration of 30% *v*/*v*, liquid-to-solid ratio of 30 mL/g, and extraction temperature of 62.7 °C. Under these conditions, the predicted extraction yields were 3.68 mg/g DW for swertiamarin and 1.62 mg/g DW for isogentisin. Experimental validation confirmed that the observed yields closely matched the predicted values (Table 3). These results demonstrate that the selected RSM models were successfully applied to optimize UAE of gentian leaves for maximal extraction of swertiamarin and isogentisin.

### 2.4. Stability of Active Ingredients

The optimal extract was used in a 30-day stability study under four different storage temperatures (−18 °C, 4 °C, 25 °C, and 40 °C) in a dark environment, while an additional set of bottles was stored at 25 °C under light. The stability of six, the most abundant active compounds (swertiamarin, gentiopicrin, mangiferin, isogentisin, isovitexin, and isoorientin) belonging to different classes of compounds (secoiridoids, flavonoids and xanthones), was monitored by comparing their residual content in the extracts, calculated as the ratio of the compound amount on the last day to that on the first day of storage, and expressed as a percentage (Table 4). The flavonoids isoorientin and isovitexin, as well as the xanthone isogentisin, were the most stable, showing no decrease in content under any storage condition. Secoiridoids also exhibited high stability, with only a slight reduction (2–4%) observed at elevated temperatures. In contrast, the xanthone mangiferin was the least stable. The most pronounced decrease in its content was observed under light exposure at room temperature (42.33%) and at elevated temperature (13.88%).

## 3. Discussion

This study supports the affirmation of leaves as an alternative medicinal source of *G. lutea*, highlighting the potential for the sustainable utilization of this endangered and protected species. A comparison of the main bioactive compounds in the roots and leaves of *G. lutea* is presented in Table 5.

### 3.1. Extraction Optimization

#### 3.1.1. Extraction of Swertiamarin

Swertiamarin, a secoiridoid glycoside commonly found in species of the Gentianaceae family, exhibits diverse pharmacological activities, including hepatoprotective, choleretic, antispasmodic, analgesic, hypoglycemic, lipid-regulating, anti-inflammatory, and immunomodulatory effects [18]. Swertiamarin was monitored as a dependent variable during the RSM-CCD optimization of UAE, as it is one of the predominant secoiridoid in the *G. lutea* leaves [11].

The reported significant positive linear effect of extraction temperature can be attributed to the disruption of cellular structures, leading to increased cell membrane permeability and enhanced diffusion of extracted compounds. However, excessively high temperatures may negatively affect thermolabile bioactive compounds (e.g., polyphenols) [17]. In the present study, within the investigated experimental range (20–80 °C), no such thermal degradation effect was observed for swertiamarin.

The most influential term in the swertiamarin model is the antagonistic interaction between ethanol concentration and extraction temperature (−BD). The practical implication of this interaction effect is that swertiamarin extraction at lower temperatures is favored by a solvent with a higher ethanol concentration, and vice versa (Figure 2c). Similarly, the reported antagonistic interaction between ethanol concentration and extraction time (−AB) indicates that shorter UAE durations benefit from higher ethanol content in the solvent, and vice versa (Figure 2a). The observed significant synergistic interaction between extraction time and temperature (+AD) is consistent with the previously reported synergistic effect between extraction time and microwave power during the optimization of microwave-assisted extraction of swertiamarin from *Swertia chirata* leaves using 50% aqueous ethanol (Figure 2b) [19]. Namely, it is well-known that microwave power and extraction temperature are closely related, given that microwave power correlates with the generated thermal energy [20]. The analyzed interaction effects highlight the advantage of RSM over the empirically most commonly used “one variable at a time” optimization approach, given that interactions cannot be assessed by conventional optimization methods.

Reports on the optimization of swertiamarin extraction from natural materials remain scarce. Optimization of the extraction technique for swertiamarin recovery from eleven *Swertia* species demonstrated that, although not the most efficient, UAE is the most economical in terms of time savings—requiring up to 30 min compared to 24 h for static extraction and continuous shaking extraction [21]. In the study by Yang et al. (2023), the UAE combined with amino acid-based deep eutectic solvents (DESs) and *β*-cyclodextrin (*β*-CD) as extraction solvents was optimized for the recovery of secoiridoids, including swertiamarin, from *Gentiana rigescens* roots and rhizomes [22]. In this study, it was reported that saturation was reached at a liquid-to-solid ratio above approximately 6 mL/g, which may explain the statistically insignificant effect of this independent variable, whose experimental range in our study was from 10 to 50 mL/g.

#### 3.1.2. Extraction of Isogentisin

Isogentisin was selected as a dependent variable during the RSM-CCD optimization of UAE, given its reported relevance to the use of *G. lutea* leaves in the treatment of angina pectoris [10]. Furthermore, its protective effects against tobacco-related diseases have been demonstrated through the reduction in cigarette smoke-induced damage in vascular endothelial cells [23].

The observed negative linear effect of ethanol concentration, identified as the most influential factor in the isogentisin model, could be explained by the greater suitability of water-rich solvents, since water acts as a plant swelling agent that facilitates deeper solvent penetration into leaf tissue [24]. At elevated ethanol concentrations, the solubility of isogentisin, being a xanthone aglycone that favors less polar solvents, becomes more pronounced, which may explain the observed positive quadratic effect of ethanol concentration. The synergistic interaction between ethanol concentration and extraction temperature (+BD) indicates that the negative effect of ethanol concentration is more pronounced at higher temperatures. The reported influence pattern of UAE process parameters on isogentisin extraction from gentian leaves differs considerably from that observed when extracting from gentian roots [16,17]. This result underscores the significant impact of matrix effects on the extractability of target compounds, suggesting that relying on empirical data from other raw materials to define optimal process parameters may lead to suboptimal results.

### 3.2. Stability Study

The stability of herbal products and extracts in terms of chemical composition is very important for preserving their biological activities. The high stability observed for both monitored flavonoids, isoorientin and isovitexin, may be attributed to their chemical structure, as they belong to the class of *C*-glycosylflavonoids, which are known to be more resistant to hydrolysis than *O*-glycosylflavonoids [25]. On the other hand, a notable difference in the stability of the monitored xanthones, isogentisin and mangiferin, was observed, which may be attributed to structural differences. Specifically, isogentisin, bearing one methoxy and two hydroxyl groups, is less reactive than mangiferin, a *C*-glucosyl xanthone with four free hydroxyl groups on the aglycone and an attached sugar moiety, making it more prone to oxidative degradation and hydrolysis. Consistent with our results, a previous study demonstrated that mangiferin is susceptible to extensive thermodegradation. Moreover, the plant extract matrix may alleviate its degradation [26]. In a study by Aberham et al. (2011) [5] investigating bioactive compounds derived from the aerial parts of *Centaurium erythraea* (a species taxonomically related to *G. lutea*) in aqueous ethanolic solution stored under various stress conditions, xanthones exhibited high stability under long-term, refrigerated, and accelerated conditions. In contrast to the pronounced stability of xanthones, the secoiridoids gentiopicrin and sweroside were susceptible to degradation under stress conditions, while swertiamarin remained relatively stable except at elevated temperatures, which led to a noticeable decline in its content [5]. Finally, the practical implication of these findings is that storage of the optimized extract at reduced temperatures (−18 °C and 4 °C in the dark), as well as at room temperature under dark conditions, may ensure acceptable preservation of the monitored bioactive compounds over a 30-day period.

## 4. Materials and Methods

### 4.1. Plant Material

The leaves of *Gentiana lutea* L. (Gentianaceae) were collected from a cultivated population (voucher No. 18070) located at a plantation in the locality of Kaluđerske Bare (1004 m a.s.l.) on Tara Mountain, Serbia. The collection was carried out in July 2024, and a voucher specimen was deposited in the Herbarium of the University of Belgrade (BEOU), Institute of Botany and Jevremovac Botanical Garden, Faculty of Biology, University of Belgrade. The plant material was air-dried in a dark, ventilated place for four days, and then ground using a laboratory mill. The powdered material was sieved through a standardized set of sieves according to the Yugoslav Pharmacopoeia (2000) [27], and the particle size fraction between 0.75 and 2 mm was used for further experiments.

### 4.2. Modeling and Optimization

Response surface methodology (RSM) employing a central composite design (CCD) was used to explore the effects of selected extraction parameters (independent variables) on the yields of target compounds (dependent variables). The investigated variables—extraction time, ethanol concentration, liquid-to-solid ratio, and extraction temperature—were tested at five levels, coded as −2, −1, 0, +1, and +2 based on the CCD matrix. The experimental plan included 29 runs, with five replicates at the central point. All runs were performed in a randomized order, as determined by the experimental design software, to minimize bias (Table 1). The selection of extraction conditions and their corresponding experimental ranges was guided by literature data on the UAE of bioactive compounds from gentian root [16]. To describe how the independent variables influence the extraction yields of swertiamarin and isogentisin, the experimental data were fitted to a second-order polynomial equation of the following form:

Y = *β*_0_ + *β*_A_A + *β*_B_B + *β*_C_C + *β*_D_D + *β*_AA_A^2^ + *β*_BB_B^2^ + *β*_CC_C^2^ + *β*_DD_D^2^ + *β*_AB_AB + *β*_AC_AC + *β*_AD_AD + *β*_BC_BC + *β*_BD_BD + *β*_CD_CD

where Y represents the response (compound yield), A is extraction time, B is ethanol concentration, C is liquid-to-solid ratio, and D is extraction temperature. The coefficient *β*_0_ is the intercept, *β*_A_, *β*_B_, *β*_C_, and *β*_D_ are the linear coefficients for the independent variables, *β*_AA_, *β*_BB_, *β*_CC_, and *β*_DD_ represent the quadratic coefficients, and the interaction effects between variables are described by *β*_AB_, *β*_AC_, *β*_AD_, *β*_BC_, *β*_BD_, and *β*_CD_.

Analysis of variance (ANOVA) was conducted to assess the statistical significance of each factor and their interactions on the responses. Variables with *p*-values below 0.05, along with those required to preserve the model hierarchy, were retained for inclusion in the model. Model adequacy was assessed through the coefficient of determination (R^2^), and statistical validation included lack-of-fit and model significance tests. To visualize the influence of the parameters, interaction and three-dimensional surface plots were generated by varying two factors while maintaining the other at its central value. The optimization of extraction parameters was performed using the desirability function approach, with all independent variables set to remain “in range” and dependent variables targeted for “maximization.” The entire experimental design, data analysis, and optimization were carried out using Design Expert 11 software (trial version, Stat-Ease Inc., Minneapolis, MN, USA).

### 4.3. Ultrasound-Assisted Extraction

Experimental runs of UAE were performed using an ultrasonic bath (Maget, Bela Palanka, Serbia) operating continuously at 35 W and 40 kHz. Ground gentian leaf samples of varying weights (0.5–2.5 g) were used to achieve liquid-to-solid ratios ranging from 10 to 50 mL/g, with the solvent volume kept constant at 25 mL. Ethanol concentrations ranged from 10 to 90% *v/v*. The extraction was carried out at temperatures between 20 and 80 °C for durations of 5 to 65 min. The Erlenmeyer flask (100 mL) containing the mixture was placed so that it was submerged 5 cm below the water surface in the ultrasonic bath. Immediately after extraction, the samples were filtered through Whatman No. 1 filter paper and subjected to chemical analysis. Each sample was filtered through 0.45 µm regenerated cellulose filter (Agilent, Santa Clara, CA, USA) and transferred into a vial prior to injection to the HPLC system. All experimental trials under the conditions defined by the RSM-CCD design were conducted as single runs.

### 4.4. HPLC Analysis

Analyses were carried out on Agilent 1200 RR HPLC system equipped with a diode array detector (DAD) and a Zorbax SB-C18 reversed-phase analytical column (Agilent, 150 mm × 4.6 mm i.d., 5 µm particle size). The mobile phase consisted of 1% *v/v* orthophosphoric acid in water (solvent A) and acetonitrile (solvent B), with a gradient elution program as follows: 98–90% A from 0 to 5 min, 90% A from 5 to 15 min, 90–85% A from 15 to 20 min, 85–70% A from 20 to 25 min, 70–40% A from 25 to 30 min, and 40–0% A from 30 to 34 min. The sample injection volume was 5 µL. The flow rate was set at 1 mL/min, and the column temperature was maintained at 25 °C. Detection was carried out at 260 and 320 nm for simultaneous identification of compounds, whereas the amounts of swertiamarin and isogentisin were determined at 260 nm. Methanolic solutions of standards were prepared to give five different concentrations for calibration curves, and concentration range was 0.1–2 mg/mL for swertiamarin and 0.03–0.5 mg/mL for isogentisin. Calibration curves were constructed by plotting the peak area vs. concentration of standard, and correlation coefficient was 0.999 for swertiamarin, and 0.996 for isogentisin. The results are presented as milligrams per gram of dry plant material (mg/g DW) [11].

### 4.5. Stability Study

Optimal extract, prepared with ethanol concentration of 30% *v*/*v*, extraction time of 50 min, liquid-to-solid ratio of 30 mL/g, and extraction temperature of 62.7 °C, was used for the stability study analysis. Clear, capped glass bottles containing the tested extract (three bottles per condition) were stored for 30 days at four different temperatures (−18 °C, 4 °C, 25 °C, and 40 °C). Samples stored at −18 °C, 4 °C, and 40 °C were kept under dark conditions, ensured by the enclosed compartments of the freezer, refrigerator, and stability chamber, respectively. At 25 °C, both light and dark conditions were simulated by storing the extract in clear glass bottles either wrapped in aluminum foil (“25 °C dark”) or left unwrapped and exposed to ambient daylight (“25 °C light”). Stability was assessed by comparing the residual content of swertiamarin, gentiopicrin, mangiferin, isogentisin, isovitexin, and isoorientin in the extracts. The results were calculated as the ratio of the compound amount determined on the first (T_0_) and last day (T_30_) of the study and expressed as a percentage (T_30_/T_0_ × 100).

## 5. Conclusions

The leaf of *Gentiana lutea* L. (Gentianaceae) has been attracting increasing interest due to its promising therapeutic potential and as an underexplored source of valuable bioactive compounds. This study supports its use as an alternative resource that may contribute to the sustainable exploitation of this overharvested species by reducing the need for root harvesting. In the present study, ultrasound-assisted extraction of bioactive compounds from *G. lutea* leaves was optimized using response surface methodology based on a central composite design. The effects of extraction time, ethanol concentration, liquid-to-solid ratio, and extraction temperature on the extraction yield of swertiamarin and isogentisin were evaluated. The influence analysis revealed a marked discrepancy in the effects of processing parameters on the yields of the target compounds. The yield of swertiamarin was primarily influenced by an antagonistic interaction between ethanol concentration and extraction temperature, whereas the yield of isogentisin was mainly dependent on ethanol concentration. The calculated optimal conditions that simultaneously maximized the extraction yield of both target compounds were 50 min of extraction time, 30% *v/v* ethanol concentration, 30 mL/g liquid-to-solid ratio, and extraction temperature of 62.7 °C. The stability study of the optimized extract demonstrated the best preservation of bioactive compounds (swertiamarin, gentiopicrin, mangiferin, isoorientin, isovitexin, and isogentisin) during storage in the dark under frozen and refrigerated conditions. Acceptable stability was also observed at room temperature in the dark. In contrast, storage at room temperature exposed to light, as well as at elevated temperature in the dark, markedly compromised mangiferin stability. The obtained results provide a solid basis for further studies focused on the separation, purification, and bioactivity assessment of selected secoiridoid and xanthone compounds. Moreover, the application of green extraction techniques such as UAE aligns with current trends toward environmentally friendly and scalable industrial processes, enhancing the feasibility of large-scale production. Overall, the conducted study supports the concept that *G. lutea* leaves may serve as a sustainable source of secoiridoid and xanthone compounds, and that the optimized extract could be further utilized in the development of value-added pharmaceutical and nutraceutical products.

## Figures and Tables

**Table 1:** Central composite design (CCD) matrix applied to ultrasound-assisted extraction of *Gentiana lutea* L. leaf bioactive compounds, with process parameters in actual and coded values as independent variables, and experimentally obtained extraction yields as dependent variables.

Std	Run	Independent Variables				Dependent Variables	
		A Extraction Time	B Ethanol Concentration	C Liquid-to-Solid Ratio	D Extraction Temperature	Swertiamarin	Isogentisin
		min	% *v*/*v*	mL/g	°C	mg/g DW *	mg/g DW
1	23	20 (−1)	30 (−1)	20 (−1)	35 (−1)	0.66	2.54
2	26	50 (+1)	30 (−1)	20 (−1)	35 (−1)	0.80	2.93
3	17	20 (−1)	70 (+1)	20 (−1)	35 (−1)	0.69	0.83
4	12	50 (+1)	70 (+1)	20 (−1)	35 (−1)	0.71	1.02
5	10	20 (−1)	30 (−1)	40 (+1)	35 (−1)	0.00	3.32
6	20	50 (+1)	30 (−1)	40 (+1)	35 (−1)	0.57	3.20
7	9	20 (−1)	70 (+1)	40 (+1)	35 (−1)	2.91	1.26
8	13	50 (+1)	70 (+1)	40 (+1)	35 (−1)	0.83	1.11
9	16	20 (−1)	30 (−1)	20 (−1)	65 (+1)	1.32	1.28
10	18	50 (+1)	30 (−1)	20 (−1)	65 (+1)	4.63	1.74
11	15	20 (−1)	70 (+1)	20 (−1)	65 (+1)	1.19	1.14
12	4	50 (+1)	70 (+1)	20 (−1)	65 (+1)	0.90	1.04
13	19	20 (−1)	30 (−1)	40 (+1)	65 (+1)	0.68	1.44
14	14	50 (+1)	30 (−1)	40 (+1)	65 (+1)	5.17	2.32
15	22	20 (−1)	70 (+1)	40 (+1)	65 (+1)	0.60	0.99
16	5	50 (+1)	70 (+1)	40 (+1)	65 (+1)	0.56	0.85
17	27	5 (−2)	50 (0)	30 (0)	50 (0)	1.20	1.42
18	11	65 (+2)	50 (0)	30 (0)	50 (0)	0.84	1.37
19	3	35 (0)	10 (−2)	30 (0)	50 (0)	0.92	2.65
20	2	35 (0)	90 (+2)	30 (0)	50 (0)	1.13	1.22
21	24	35 (0)	50 (0)	10 (−2)	50 (0)	0.93	1.16
22	28	35 (0)	50 (0)	50 (+2)	50 (0)	0.59	1.23
23	7	35 (0)	50 (0)	30 (0)	20 (−2)	0.84	1.91
24	25	35 (0)	50 (0)	30 (0)	80 (+2)	0.86	1.10
25	1	35 (0)	50 (0)	30 (0)	50 (0)	1.29	1.39
26	21	35 (0)	50 (0)	30 (0)	50 (0)	1.31	1.47
27	29	35 (0)	50 (0)	30 (0)	50 (0)	1.34	1.42
28	6	35 (0)	50 (0)	30 (0)	50 (0)	0.74	1.32
29	8	35 (0)	50 (0)	30 (0)	50 (0)	0.67	1.20

* DW—dry weight of plant material.

**Table 2:** Analysis of variance (ANOVA) results for the fitted second-order polynomial models of the examined parameters.

Terms	*p*-Value	
	Swertiamarin	Isogentisin
Linear		
A—Extraction time	0.1213	/
B—Ethanol concentration	0.1498	<0.0001 ***
C—Liquid-to-solid ratio	/	/
D—Extraction temperature	0.0275 *	0.0002 ***
Interaction		
AB	0.0006 ***	/
AC	/	/
AD	0.0038 **	/
BC	/	/
BD	0.0003 ***	0.0028 **
CD	/	/
Quadratic		
A^2^	/	/
B^2^	/	0.0237 *
C^2^	/	/
D^2^	/	/
Model fitting assessment		
Model	<0.0001 ***	<0.0001 ***
R^2^	0.7143	0.8429
Lack-of-fit	0.0671	0.0619

/—Term not included in the final model; level of significance: * 0.01 < *p* < 0.05; ** 0.001 < *p* < 0.01; *** *p* < 0.001.

**Table 3:** Confirmation results of the model with the measured values and the predicted values of the studied responses.

Response Values	Predicted Content (mg/g DW)	Measured Content (mg/g DW *)
Swertiamarin	3.68	3.75 ± 0.19
Isogentisin	1.62	1.57 ± 0.08

* DW—dry weight of plant material.

**Table 4:** Residual content of bioactive compounds from *Gentiana lutea* L. leaves during 30-day storage under various conditions.

Sample	Swertiamarin	Gentiopicrin	Mangiferin	Isoorientin	Isovitexin	Isogentisin
−18 °C dark	100.2 ± 0.2%	100.1 ± 0.2%	99.9 ± 0.2%	100.1 ± 0.2%	100.1 ± 0.1%	100.3 ± 0.2%
4 °C dark	100.2 ± 0.2%	100.2 ± 0.2%	100.0 ± 0.3%	100.1 ± 0.1%	100.1 ± 0.2%	100.2 ± 0.2%
25 °C dark	100.1 ± 0.2%	100.2 ± 0.1%	94.5 ± 0.2%	100.2 ± 0.2%	100.2 ± 0.3%	100.1 ± 0.2%
25 °C light	100.4 ± 0.2%	100.3 ± 0.2%	57.7 ± 0.9%	100.2 ± 0.3%	100.2 ± 0.2%	99.9 ± 0.3%
40 °C dark	98.2 ± 0.5%	96.3 ± 0.4%	86.1 ± 0.3%	99.9 ± 0.2%	99.8 ± 0.4%	100.1 ± 0.2%

**Table 5:** Comparison of the main bioactive compounds in the roots and leaves of *Gentiana lutea* L. [7].

Class of Phytocompounds	*G. lutea* Roots	*G. lutea* Leaves
Iridoids and secoiridoids	Loganic Acid Sweroside Swertiamarin Gentiopicrin Amarogentin	Swertiamarin Gentiopicrin Eustomoside Eustomorusside Septemfidoside
Flavonoids	/	Isovitexin Isosaponarin Isoorientin Isoorientin 2″-*O*-glucoside Isoorientin 4′-*O*-glucoside
Xanthones	Gentioside Gentisin Isogentisin	Mangiferin Isogentisin

## Data Availability

Data are contained within the article.

## References

[1] Jiang M., Cui B.W., Wu Y.L., Nan J.X., Lian L.H. (2021). Genus *Gentiana*: A review on phytochemistry, pharmacology and molecular mechanism. J. Ethnopharmacol..

[2] Pan Y., Zhao Y.L., Zhang J., Li W.Y., Wang Y.Z. (2016). Phytochemistry and Pharmacological Activities of the Genus *Gentiana* (Gentianaceae). Chem. Biodivers..

[3] Pasdaran A., Butovska D., Kerr P., Naychov Z., Aneva I., Kozuharova E. (2022). Gentians, natural remedies for future of visceral pain control: An ethnopharmacological review with an in silico approach. Biol. Futur..

[4] Carnat A., Fraisse D., Carnat A.P., Felgines C., Chaud D., Lamaison J.L. (2005). Influence of drying mode on iridoid bitter constituent levels in gentian root. J. Sci. Food Agric..

[5] Aberham A., Pieri V., Croom E.M., Ellmerer E., Stuppner H. (2011). Analysis of iridoids, secoiridoids and xanthones in *Centaurium erythraea*, *Frasera caroliniensis* and *Gentiana lutea* using LC–MS and RP-HPLC. J. Pharm. Biomed. Anal..

[6] Jovanović M., Ćujić-Nikolić N., Drinić Z. (2021). Spray drying of *Gentiana asclepiadea* L. root extract: Successful encapsulation into powders with preserved stability of bioactive compounds. Ind. Crops Prod..

[7] Ponticelli M., Lela L., Moles M., Mangieri C., Bisaccia D., Faraone I., Falabella R., Milella L. (2023). The healing bitterness of *Gentiana lutea* L., phytochemistry and biological activities: A systematic review. Phytochemistry.

[8] European Medicines Agency Assessment Report on *Gentiana lutea* L., *radix*. Ref.: EMA/HMPC/607863/2017. https://www.ema.europa.eu/en/documents/herbal-report/assessment-report-gentiana-lutea-l-radix-revision-1_en.pdf.

[9] Fiorito S., Epifano F., Palumbo L., Collevecchio C., Mascioli F., Spogli R., Genovese S. (2022). Leaves of Yellow Gentian (*Gentiana lutea*) as an Alternative Source of Bitter Secoiridoid Glycosides. J. Nat. Prod..

[10] Janković T., Mudrić J., Radojičić V., Pejić L., Nikolić N.Ć., Marković S., Menković N. (2025). Smoking of *Gentiana lutea* leaves: Validation of its traditional use. J. Pharm. Biomed. Anal..

[11] Balijagić J., Janković T., Zdunić G., Bošković J., Šavikin K., Gođevac D., Stanojković T., Jovančević M., Menković N. (2012). Chemical Profile, Radical Scavenging and Cytotoxic Activity of Yellow Gentian Leaves (*Genitaneae luteae folium*) Grown in Northern Regions of Montenegro. Nat. Prod. Commun..

[12] Menković N., Šavikin-Fodulović K., Savin K. (2000). Chemical Composition and Seasonal Variations in the Amount of Secondary Compounds in *Gentiana lutea* Leaves and Flowers. Planta Med..

[13] Oubannin S., Bijla L., Ahmed M.N., Ibourki M., El Kharrassi Y., Devkota K., Bouyahya A., Maggi F., Caprioli G., Sakar E.H. (2024). Recent advances in the extraction of bioactive compounds from plant matrices and their use as potential antioxidants for vegetable oils enrichment. J. Food Compos. Anal..

[14] Kumar K., Srivastav S., Sharanagat V.S. (2021). Ultrasound assisted extraction (UAE) of bioactive compounds from fruit and vegetable processing by-products: A review. Ultrason. Sonochem..

[15] Yusoff I.M., Taher Z.M., Rahmat Z., Chua L.S. (2022). A Review of ultrasound-assisted extraction for plant bioactive compounds: Phenolics, flavonoids, thymols, saponins and proteins. Food Res. Int..

[16] Živković J., Janković T., Menković N., Šavikin K. (2019). Optimization of ultrasound-assisted extraction of isogentisin, gentiopicroside and total polyphenols from gentian root using response-surface methodology. Ind. Crops Prod..

[17] Mudrić J., Janković T., Šavikin K., Bigović D., Đukić-Ćosić D., Ibrić S., Đuriš J. (2020). Optimization and modelling of gentiopicroside, isogentisin and total phenolics extraction from *Gentiana lutea* L. Roots. Ind. Crops Prod..

[18] Shi M., Tang J., Zhang T., Han H. (2022). Swertiamarin, an active iridoid glycoside from *Swertia pseudochinensis* H. Hara, protects against alpha-naphthylisothiocyanate-induced cholestasis by activating the farnesoid X receptor and bile acid excretion pathway. J. Ethnopharmacol..

[19] Kaur P., Pandey D.K., Gupta R.C., Dey A. (2019). Simultaneous microwave assisted extraction and HPTLC quantification of mangiferin, amarogentin, and swertiamarin in *Swertia* species from Western Himalayas. Ind. Crops Prod..

[20] Dao T.A.T., Webb H.K., Malherbe F. (2021). Optimization of pectin extraction from fruit peels by response surface method: Conventional versus microwave-assisted heating. Food Hydrocoll..

[21] Kshirsagar P.R., Gaikwad N.B., Pai S.R., Bapat V.A. (2017). Optimization of extraction techniques and quantification of swertiamarin and mangiferin by using RP-UFLC method from eleven *Swertia* species. S. Afr. J. Bot..

[22] Yang Y., Zhao R., Gao H., Wang Z., Yang X., Ruan M., Gu H., Yang L., Tian H., Fan C. (2023). β-Cyclodextrin as a booster for ultrasound-assisted extraction of secoiridoids from *Gentiana rigescens* using a biobased deep eutectic solvent. Ind. Crops Prod..

[23] Schmieder A., Schwaiger S., Csordas A., Backovic A., Messner B., Wick G., Stuppner H., Bernhard D. (2007). Isogentisin—A novel compound for the prevention of smoking-caused endothelial injury. Atherosclerosis.

[24] Şahin S., Şamlı R. (2013). Optimization of olive leaf extract obtained by ultrasound-assisted extraction with response surface methodology. Ultrason. Sonochem..

[25] Zeng P., Zhang Y., Pan C., Jia Q., Guo F., Li Y., Zhu W., Chen K. (2013). Advances in studying of the pharmacological activities and structure-activity relationships of natural *C*-glycosylflavonoids. Acta Pharm. Sin. B.

[26] Beelders T., de Beer D., Kidd M., Joubert E. (2018). Modeling of thermal degradation kinetics of the *C*-glucosyl xanthone mangiferin in an aqueous model solution as a function of pH and temperature and protective effect of honeybush extract matrix. Food Res. Int..

[27] (2000). Yugoslavian Pharmacopeia (Pharmacopoea Jugoslavica).

